# Effects of postural threat on perceptions of lower leg somatosensory stimuli during standing

**DOI:** 10.3389/fnins.2023.1191976

**Published:** 2023-08-09

**Authors:** Taylor W. Cleworth, Ryan M. Peters, Romeo Chua, J. Timothy Inglis, Mark G. Carpenter

**Affiliations:** ^1^School of Kinesiology and Health Science, York University, Toronto, ON, Canada; ^2^Centre for Vision Research, York University, Toronto, ON, Canada; ^3^Faculty of Kinesiology, University of Calgary, Calgary, AB, Canada; ^4^Hotchkiss Brain Institute, University of Calgary, Calgary, AB, Canada; ^5^School of Kinesiology, University of British Columbia, Vancouver, BC, Canada; ^6^International Collaboration for Repair Discoveries, University of British Columbia, Vancouver, BC, Canada; ^7^Djavad Mowafaghian Centre for Brain Health, University of British Columbia, Vancouver, BC, Canada

**Keywords:** postural threat, balance, perception, somatosensory, fear, anxiety

## Abstract

Height-induced postural threat affects emotional state and standing balance behaviour during static, voluntary, and dynamic tasks. Facing a threat to balance also affects sensory and cortical processes during balance tasks. As sensory and cognitive functions are crucial in forming perceptions of movement, balance-related changes during threatening conditions might be associated with changes in conscious perceptions. Therefore, the purpose of this study was to examine the changes and potential mechanisms underlying conscious perceptions of balance-relevant information during height-induced postural threat. A combination of three experimental procedures utilized height-induced postural threat to manipulate emotional state, balance behavior, and/or conscious perceptions of balance-related stimuli. Experiment 1 assessed conscious perception of foot position during stance. During continuous antero-posterior pseudorandom support surface rotations, perceived foot movement was larger while actual foot movement did not change in the High (3.2 m, at the edge) compared to Low (1.1 m, away from edge) height conditions. Experiment 2 and 3 assessed somatosensory perceptual thresholds during upright stance. Perceptual thresholds for ankle rotations were elevated while foot sole vibrations thresholds remained unchanged in the High compared to Low condition. This study furthers our understanding of the relationship between emotional state, sensory perception, and balance performance. While threat can influence the perceived amplitude of above threshold ankle rotations, there is a reduction in the sensitivity of an ankle rotation without any change to foot sole sensitivity. These results highlight the effect of postural threat on neurophysiological and cognitive components of balance control and provide insight into balance assessment and intervention.

## 1. Introduction

Postural threat associated with standing on elevated surfaces, or anticipation of a balance perturbation affects emotional state and standing balance behavior during static, voluntary, and dynamic tasks (Adkin and Carpenter, 2018). Facing a threat to balance also affects sensory and cortical processes during balance tasks (Adkin et al., 2008; Davis et al., 2011; Horslen et al., 2013; Naranjo et al., 2016). Fear and anxiety responses to actual or perceived threats can be accompanied by changes in perception of individual sensory inputs, including auditory (Borsky, 1979; Siegel and Stefanucci, 2011; Asutay and Västfjäll, 2012; Gagnon et al., 2013), visual (Stefanucci et al., 2008; Teachman et al., 2008; Clerkin et al., 2009; Vasey et al., 2012) and tactile stimuli (Shi et al., 2012). Multi-sensory perceptions, such as those related to whole-body postural sway (Fitzpatrick and McCloskey, 1994; Mergner and Rosemeier, 1998), have also been shown to be amplified under threatening conditions during quiet standing (Cleworth and Carpenter, 2016), voluntary leaning (Cleworth et al., 2018), and dynamic stance tasks (Cleworth et al., 2019). However, the mechanisms through which fear and anxiety affect these perceptions of whole-body movement are currently not known.

Changes in the amplitude of perceived movements may be mediated by (a) a decrease in the detectable threshold (stimulus/contrast gain), (b) an amplification of the response proportional to stimulus intensity (response gain), or (c) a combination of the two (Horak and Diener, 1994; Lim et al., 2014). Decreasing the threshold for detectable movements will allow smaller movements to be perceived, increasing somatosensory acuity. Alternatively, the response to a detectable stimulus may be modified by a gain factor resulting in an increased response strength proportional to stimulus intensity, independent of changes in perceptual thresholds.

Both stimulus gain and response gain may rely on peripheral sensory receptors and central processing of the sensory information (Dannenbaum and Jones, 1993; Simoneau et al., 1995), both of which have been shown to be affected by a height-induced postural threat (Adkin et al., 2008; Sibley et al., 2010; Davis et al., 2011; Horslen et al., 2013; Naranjo et al., 2016). Whole-body sway is thought to be heavily reliant on afferent information from somatosensory receptors in the ankle (Thelen et al., 1998), as the majority of sway occurs about the ankle joint in the sagittal plane (Gage et al., 2004). Likewise, movement detection thresholds are lowest for somatosensory-related movements compared to visual or vestibular systems (Fitzpatrick and McCloskey, 1994). It is therefore important to consider the somatosensory system as a potential contributor to height-related effects on perception.

The aim of this study was to investigate the effect of height-induced postural threat on lower leg somatosensory acuity. Since somatosensory acuity relies on different classes of somatosensory receptors including muscle spindles and cutaneous mechanoreceptors (Refshauge and Fitzpatrick, 1995), multiple experiments were needed to determine how different classes of somatosensory receptors contribute to any height-related changes. Three unique experiments were performed to examine how threat influences conscious perception of passive ankle rotation (Experiment 1), and perceptual thresholds for passive ankle rotation and cutaneous foot sole stimulation (Experiments 2 and 3 respectively) in a fully loaded leg during upright stance. Uni-lateral stimuli (ankle rotations and foot-sole stimulation) were applied with subjects braced to minimize balance perturbations, and isolate perception of somatosensory changes from other potential sensory inputs (ie vestibular) that would be involved in whole-body sway. Based on prior work, we hypothesized that conscious perception and perceptual thresholds of passive ankle joint rotation would be larger under postural threat and independent of rotation direction (Adkin et al., 2008; Davis et al., 2011; Horslen et al., 2013, 2018; Cleworth and Carpenter, 2016; Cleworth et al., 2019). Likewise, we hypothesized that height-induced threat would decrease perceptual thresholds of foot-sole vibrations independent of frequency, while higher frequency vibrations would be more easily perceived than low frequency across height conditions (Strzalkowski et al., 2015).

## 2. Materials and methods

A total of 45 young healthy adults volunteered to participate in this study across 3 experiments; Experiment 1: *n* = 14 (9 Female), mean age 25.4 ± 5.0 years; Experiment 2: *n* = 15 (9 Female), mean age 26.5 ± 3.8 years; Experiment 3: *n* = 18 (10 Female), mean age 27.2 ± 5.2 years. All participants self-reported having no known neurological, orthopedic, or cognitive disorders that may affect their balance performance, perception of ankle rotations or foot sole vibrations. The University of British Columbia Clinical Research Ethics Board approved the experimental procedures. All methods were performed in accordance with the relevant guidelines and regulations at UBC, and in accordance with the Declaration of Helsinki. All participants gave informed written consent prior to participation.

### 2.1. Common methods for all 3 experiments:

**Figure 1:**
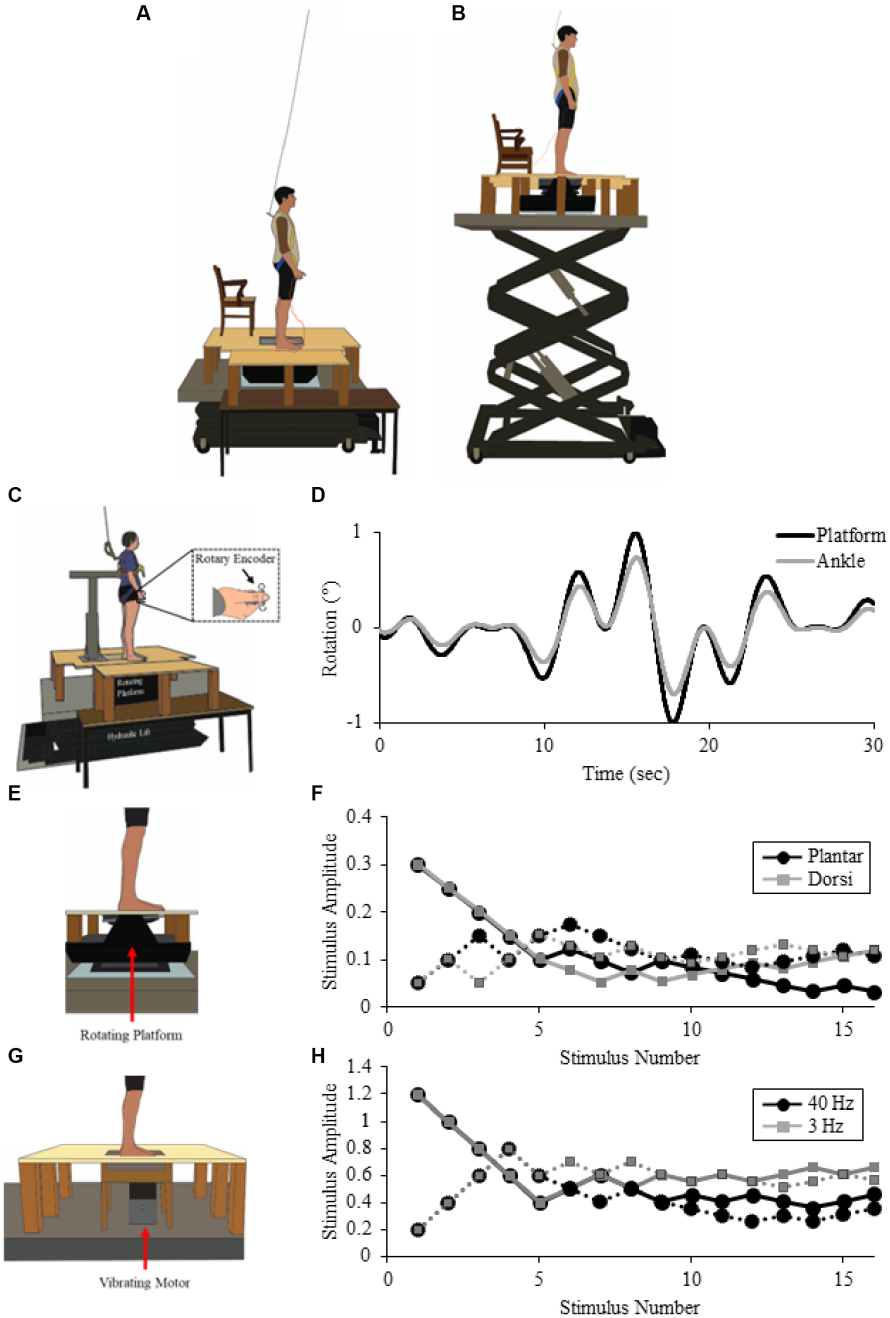
Experimental setup. Illustration of experimental setup for Low **(A)** and High **(B)** conditions (from Experiment 2). Also, illustration of experimental setup for the Low condition in Experiment 1 **(C)**, Experiment 2 **(E)** and Experiment 3 **(G)**. Participants stood on a force plate on top of a rotating platform **(C,E)**, Experiment 1 or 2, respectively or force platform with a vibrating probe protruding through the support surface [**(G)**, Experiment 3]. Representative data for Experiment 1 [**(D)**; platform (black) and Ankle (gray) displacement], Experiment 2 [**(F)**; ascending (dotted line) and descending (solid line) staircase for plantar- (black) and dorsi-flexion (gray) rotations], and Experiment 3 [**(H)**; ascending (dotted line) and descending (solid line) staircase for 40 Hz (black) and 3 Hz (gray) vibrations].

In all experiments, participants stood under different conditions of threat manipulated by adjusting the surface height on which they stood using a hydraulic lift (M419-207B01H01D, Pentalift, Canada). In the Low threat condition, the top of the standing support surface was 1.1 m above the ground. An extension was added to the right of the participant 60 cm beyond the edge of the platform to position the participants further away from the edge of the support surface, providing a stable surface in all directions, and further reduce threat in this condition (Figure 1A; Carpenter et al., 2001). In the High threat condition, the support surface was raised to 3.2 m above the ground, with no additional support surface to the right of the participant to maximize threat effects (Figure 1B; Carpenter et al., 2001).

Emotional responses to the threat manipulation were confirmed using self-reported questionnaires, and physiological measures of arousal. Prior to each experimental condition, seated participants rated how confident they felt they could remain upright and avoid a fall on a scale from 0 (not confident at all) to 100 (completely confident). After each condition, participants provided a subjective rating of fear of falling (0 = no fear, 100 = fearful), perceived stability (0 = not stable, 100 = very stable), and state anxiety using a 16 item, 9-point Likert scale assessing elements of somatic, worry and concentrations that were summed to a total anxiety score (Hauck et al., 2008). Electrodermal activity (EDA) was collected from the thenar and hypothenar eminences of the left hand (100 Hz, Skin Conductance Module 2502, Cambridge Electronic Design, UK) as a physiological measure of sympathetic arousal (Critchley, 2002).

In all experiments, participants stood with their eyes open, and gaze fixated on a visual target located approximately 3 meters away at eye level. Feet were positioned side-by-side with a maximum distance of 40 cm. In Experiment 1 & 2, the left foot was positioned on a AMTI force plate (model OR6-7-1000, AMTI, United States), while in Experiment 3, the left foot was positioned on a custom force plate embedded with 4 vertical load cells (Experiment 3; SSB-250 with BSC4A-C14, Interface Advanced Force Measurement, United States). Vertical ground reaction forces were sampled at 100 Hz. Prior to the first experimental condition, participants stood quietly for 20 s to establish pre-stimulus baseline (neutral) measures. Baseline vertical force level was monitored throughout experimental trials and used to provide verbal feedback from the experimenter if a neutral position was deviated from by two standard deviations. For Experiment 2 and 3, feedback was delivered when necessary, only immediately after a stimulus was perceived to minimize the possibility of shifting attention away from the psychophysical task.

3D motion capture data were collected to estimate platform (Experiment 1 and 2) and lower limb position (all experiments, 250 Hz, accuracy: 0.1 mm, resolution: 0.01 mm, Optotrak, Northern Digital Inc.). Infrared emitting diodes were placed at the front and back of the force plate’s top surface, left base of the fifth metatarsal, lateral malleolus, and fibular head. Two-dimensional filtered coordinates defining the foot (toe to lateral malleolus) and shank (malleolus to fibular head) were used to calculate ankle angle.

Electromyography (EMG) was recorded using a bipolar arrangement of 2 surface electrodes placed 2 cm apart over the muscle bellies of the left soleus (SOL) and tibialis anterior (TA). EMG was band-pass filtered between 10 and 500 Hz (Telemyo, 2400R, Noraxon, United States), and sampled at 2000 Hz. Offline, EMG data was low-pass filtered at 100 Hz using a dual-pass Butterworth filter, bias corrected, and full-wave rectified. Mean background EMG activity, vertical force, and foot and ankle position were calculated offline from the entire trial (Experiment 1) or from 1 s prior to stimulus onset (Experiment 2 and 3) in each height condition.

### 2.2. Experiment-specific methods and procedures

#### 2.2.1. Experiment 1

Participants stood barefoot while strapped to a rigid structure (see Figure 1C) limiting the amount of anteroposterior (AP) sway (Lackner, 2021). In addition, reducing the amount of AP sway allowed for a consistent stimulus across height conditions as postural threat can change sway amplitude and leaning (Adkin and Carpenter, 2018) which could otherwise influence the amount of ankle rotation. The force plate under the left foot was mounted to a custom-built single axis servo-controlled tilting platform; the right foot was positioned on an adjacent stable surface. The feet were positioned so that the axis of rotation of the tilting platform was aligned with the participant’s ankle joints. Foot position was marked on the force plate and kept constant across trials to ensure a consistent rotation of the ankle. During experimental conditions, participants were instructed to remain upright and avoid using the brace as support during continuous AP support surface oscillations of the left ankle. During continuous support surface rotations used to induce small and large movements (pseudorandom oscillations <0.5 Hz, ± 1°, see Figure 1C and Figure 1D), participants were instructed to track accurately their ankle position in real-time using a hand-held rotary encoder (1/4″ shaft, model E14102402302, Dynapar, United States). Two weak springs, one on either side of the point of contact with the thumb were used to limit the amount of drift that could occur using the tracking device (Cleworth and Carpenter, 2016; Cleworth et al., 2019), and provided minimal feedback of the encoder’s neutral (vertical) position. Participants performed a minimum of two practice trials, lasting 30 s each, to become familiar with the ankle movements and to practice using the device. Participants completed the practice trials first with eyes open and then eyes closed. The experimenter ensured proper use of the device during these trials by comparing ankle angular displacement, platform angular displacement, and tracked position (rotary encoder voltage). If ankle rotation and tracked position were congruent in amplitude and direction (visual inspection for similar patterns), and participants reported ease of use, the experiment continued (all participants correctly performed the task within two eyes open and one eyes closed condition). Participants then performed two seven-minute trials in the Low condition (to control for first trial effects, while the second trial was used in the analysis), and one in the High condition with their eyes open.

##### 2.2.1.1. Analysis

The rotary encoder voltages and ground reaction forces and moments were collected and exported at 2000 Hz (Power 1401 with Spike2 software, CED, UK). Tracked sway was determined from the voltage of the rotary encoder and was band-pass filtered using a 0.005 Hz to 2 Hz dual-pass Butterworth filter (Cleworth et al., 2019). For ankle and tracked data, the mean position was calculated and subtracted from each respective trace to remove any bias. Due to the tracked sway data having a ‘unitless’ quantity, data were normalized to the Low condition and expressed as a percentage of this movement (Cleworth et al., 2019). Normalization was calculated by dividing each data point by the maximum value in the Low condition. Both the Low and High condition data were normalized to the maximum amplitude from the Low condition.

Root mean square (RMS) were calculated from platform, ankle and tracked data in the AP direction from the unbiased normalized signal to quantify the amplitude of actual and perceived movement. A quotient (QRMS) was then calculated between perceived and actual movement (tracked RMS was divided by ankle RMS) to signify the relative changes in perceived movement related to actual movement within a condition. Three participants were removed prior to analysis due to an inability to perform the task correctly (difficulty in tracking, an inability to remain static during continuous rotations, or could not complete the task).

Cross correlation analyses were performed to quantify the participants’ ability to track ankle rotation. The time (lag) and amplitude of the maximum cross-correlation coefficient with a maximum lag of 1.5 s were calculated from the ankle and tracked data in the AP direction from the unbiased normalized signal for both height conditions.

#### 2.2.2. Experiment 2

Similar to Experiment 1, participants stood barefoot with the left foot on a force plate mounted to a rotating motorized platform, right foot on an adjacent stable surface, and both ankle joints aligned with the platform’s axis of rotation (Figure 1E). While standing in the two postural threat conditions, participants performed an ankle rotation discrimination task. The left ankle was rotated in the pitch plane in a dorsi-flexion or plantar-flexion direction. Platform rotation speed was kept constant at 0.25°/s while amplitude was varied. Potentiometer-based feedback from the platform and the signal supplying the motor was recorded (sampled at 2000 Hz, Power 1401 with Spike2 software, CED, UK). Participants were asked to indicate when they felt either an ankle rotation by pushing a right hand-held bidirectional toggle switch either up for dorsi-flexion or down for plantar-flexion. The correct detection of an ankle rotation was only accepted if the switch was correctly pushed within 2 s after platform movement offset. To reduce the likelihood of false-positive responses, participants were specifically instructed to indicate a movement direction only when they were sure they had felt a movement, and to not respond if no movement or an unidentifiable movement was perceived.

Prior to any experimental conditions, participants completed two practice trials to become familiar with the experimental procedures, and to remove any first trial effects. At Low height, participants were asked to identify which of two large amplitude ankle rotations they felt (using the toggle switch) followed by a verbal report. A minimum of five randomly ordered stimuli were administered using a constant amplitude (all presumed suprathreshold; 1° rotation). This trial was used to ensure proper use of experimental equipment, and that each participant could detect large amplitude stimuli. The second practice trial consisted of 16 stimuli of each direction, randomly presented to ensure participants could sufficiently perform the task.

When assessing the sensitivity within the proprioceptive system, there are a number of methods that can be used to determine a threshold, including method of limits, constant stimuli, and adaptive staircase methods. Due to the time limitations for maintaining emotional changes with height, an adaptive staircase method was selected because of its relatively short duration (5 min) compared to approximately 20 min needed for a method of limits approach (Berquin et al., 2010). Between stimuli, the peak-to-peak amplitude of the voltage command was adjusted using an adapted staircase method (4–2-1-step, modified from Dyck et al., 1993). Each staircase starts with 0.1 V (approximately 0.2° which is above thresholds previously determined, Fitzpatrick and McCloskey, 1994) steps in stimulus intensity. Steps were halved to 0.05 V after four steps. Stimuli were halved again to 0.025 V after another four steps and kept constant for seven steps. This method allowed for stimuli to remain within the motion capture systems ability to detect stimuli amplitudes, and provided more efficient (fewer trials) thresholds in pilot data than the reduction in step size based on reversals as previously used (Dyck et al., 1993; Peters et al., 2016, where a reversal point, when the participant goes from responding “yes” to responding “no,” or vice versa, reduced the step size by half until the next reversal point, reducing by half again until all trials are given). To accommodate the discrimination technique, two staircases were interlaced, one for each of the two stimuli (Figure 1F). Furthermore, to accommodate an ascending and descending staircase method (typically seen in a method of limits technique), a new staircase was implemented halfway through each experimental condition. Staircase-direction was counterbalanced across participants such that stimulus 17 for each of the movement directions was set at a low or high amplitude depending on whether the start of the experiment started with a high or low amplitude, respectively. The 4–2-1-step algorithm was then reset and continued until the end of the block of trials. Stimulus-direction was randomly presented to avoid any anticipation bias.

##### 2.2.2.1. Analysis

To compute ankle rotation discrimination thresholds, data were reanalyzed offline using the peak displacement of the foot calculated from motion capture (see limitations). In accordance with previous work (Peters et al., 2016), this method accounted for trial-to-trial variability and inter-individual differences in biomechanical properties of the ankle and/or foot. Thresholds were then calculated as the mean of the smallest step-amplitude reversal points. If only one reversal point for a given direction was observed in the smallest step-amplitudes, the last two stimuli were averaged and used to calculate a mean. False detection rates were calculated from all 2 s periods where no platform movement was given within a trial and reported as a percentage of the number of times a stimulus was perceived during these periods (number of blank stimuli detected divided by total number of 2 s periods with no stimulus). Participants were removed from further analysis if a false detection rate in the Low condition exceeded 20% (Berquin et al., 2010). As a result, one participant was removed due to large false detection rates. One additional outlier was removed due to higher than normal (two times higher than any other participant) thresholds within the Low condition.

#### 2.2.3. Experiment 3

A 6 mm probe protruded through a 7 mm opening in the customized force plate and contacted the left foot sole (Figure 1G). The left foot was positioned to align to the probe with a location approximately 80% of maximum width from the lateral border near the ball of the foot, and 80% of maximum length from the tip of the big toe to the back of the heel. If the probe aligned with the space between the metatarsal and phalange, where there is little to no skin contact with the support surface, the foot was moved anteriorly until sufficient force (approximately 2 N) on the probe was obtained (less than 5 mm). Corresponding with previous studies (Peters et al., 2016), this location corresponded to the skin over the anterior aspect of the metatarsal head, which has been reported to be more tightly coupled to balance relative to more posterior areas of skin on the sole of the foot in the elderly (Cruz-Almeida et al., 2014). Foot position was marked on the force plate and kept constant across trials to ensure a consistent contact force of the probe onto the skin surface (Table 1). The probe was attached to a linear motor (model MT-160; Labworks) in series with a force transducer (model 31; Honeywell). An accelerometer (model 2220–010; X Tronics) was also secured to the back of the motor piston. Acceleration and force from the single force transducer were differentially amplified (×1 and ×100, respectively) and online low-pass filtered at 600 Hz (Brownlee model 440; AutoMate Scientific), and sampled at 5 kHz (Power 1401 with Spike2 software, CED, UK).

**Table 1:** Summary of statistical test results for emotional state and baseline results.

	Experiment 1				Experiment 2				Experiment 3			
	Low x̄ SD	High x̄ SD	t(11) p	d	Low x̄ SD	High x̄ SD	t(12) p	d	Low x̄ SD	High x̄ SD	t(16) p	*d*
Emotional state measures												
Balance confidence	**96.36** **6.74**	**80.91** **20.83**	**2.974 0.013**	**0.86**	**99.23** **2.77**	**80.23** **19.77**	**3.672** **0.003**	**1.02**	**95.56** **7.05**	**80.56** **19.62**	**3.112** **0.007**	**0.75**
Stability	**90.45** **11.30**	**66.64** **30.06**	**2.965 0.013**	**0.86**	**88.31** **12.89**	**75.38** **20.86**	**2.403** **0.033**	**0.66**	**90.83** **11.01**	**71.94** **21.22**	**4.261** **0.001**	**1.03**
Fear	**3.45** **5.13**	**21.00** **21.29**	**3.060 0.011**	**0.88**	**1.62** **3.73**	**22.31** **17.27**	**4.341** **0.001**	**1.20**	**4.72** **7.57**	**29.17** **24.15**	**5.088** **< 0.001**	**1.23**
Anxiety	**33.91** **15.24**	**47.36** **21.01**	**3.001 0.012**	**0.87**	**24.23** **10.61**	**38.46** **21.92**	**2.710** **0.019**	**0.75**	**24.39** **6.27**	**42.83** **17.78**	**5.270** **< 0.001**	**1.28**
EDA	**11.85** **7.93**	**14.89** **7.73**	**3.604 0.004**	**1.04**	**20.70** **4.21**	**23.82** **4.20**	**3.420** **0.005**	**0.95**	**19.84** **10.73**	**27.09** **15.47**	**2.959** **0.009**	**0.72**
Baseline measures												
Vertical force	**1.31** **0.28**	**1.37** **0.29**	**2.541** **0.028**	**0.73**	**1.10** **0.41**	**1.14** **0.43**	**3.467 0.004**	**0.99**	**2.63** **0.55**	**2.72** **0.58**	**3.722** **0.002**	**0.90**
SOL BGD	7.8 4.1	8.1 4.4	1.54 0.152	0.44	6.7 4.2	7.1 4.6	0.638 0.535	0.18	8.7 3.3	8.8 3.0	0.233 0.818	0.06
TA BGD	**2.6** **2.7**	**2.7** **2.8**	**2.237** **0.047**	**0.65**	1.3 1.0	1.7 1.4	1.141 0.275	0.32	1.5 1.5	5.0 8.9	1.863 0.081	0.45
Pre-stim foot POS					0.02 0.07	0.02 0.32	0.389 0.704	0.11	<0.001 0.014	0.002 0.005	0.511 0.617	0.12
Pre-stim ankle angle					100.9 34.54	100.6 34.45	0.965 0.352	0.27	62.74 2.83	63.12 2.79	1.705 0.108	0.41
Pre-stim probe force									5.35 0.96	5.41 1.04	0.833 0.417	0.20

Significant effects are in bold. Gray filled areas indicate variables not measured for the experiment.

x̄ = mean; SD = standard deviation; t(#) = t statistic with degrees of freedom; *p* = *p* value; *d* = Cohen’s d; EDA = electrodermal activity; SOL = Soleus; BGD = background TA = tibialis anterior; POS = position; pre-stim = pre-stimulus.

During upright stance, vibrations were applied at random intervals (3 to 5 s) perpendicular to the skin. Vibration stimuli were applied for 1 s in duration. Participants were asked to indicate when they felt either a 3 Hz vibration by pushing a button in one hand, or a 40 Hz vibration by pushing a button the other hand (participants were able to choose which hand held the 3 and 40 Hz buttons, and this orientation was kept constant across height conditions). The correct detection of a vibration was only accepted if the button was pushed between vibration onset and 1 s after vibration offset (2 s total). To reduce the likelihood of false-positive responses, participants were specifically instructed to push a button (indicating 3 Hz or 40 Hz felt) only when they were sure they had felt the given vibration, and to not respond if no vibration or unidentifiable vibration was felt.

Prior to any experimental conditions, participants completed two practice trials to familiarize with the experimental procedures, and to remove any first trial effects. At Low height, participants were asked to identify which of two foot sole vibrations they felt (using the correct input device for each stimulus) followed by a verbal report. A minimum of five randomly ordered stimuli were administered using a constant amplitude (all presumed suprathreshold; 3 Hz >1 N and 40 Hz > 0.5 N). This trial was used to ensure proper use of experimental equipment, and that each participant could detect large amplitude stimuli. The second practice trial consisted of 16 stimuli of each frequency, randomly presented to ensure participants could sufficiently perform the task.

Similar to Experiment 2, during the experimental trials, the peak-to-peak amplitude of the voltage command was adjusted from stimulus-to-stimulus using an adapted staircase method (4–2-1-step, modified from Dyck et al., 1993), and staircases (Figure 1H) for both frequencies were interlaced. Descending staircases were used prior to ascending for all participants to ensure suprathreshold stimuli were used first. Stimulus frequency was randomly presented to avoid any anticipation bias.

##### 2.2.3.1. Analysis

A similar method to Experiment 2 was used to compute discrimination thresholds for foot sole vibrotactile sensation using peak-to-peak force amplitude from the force transducer. No participants were removed due to large false detection rates, but one outlier was removed due to higher than normal (two times higher than any other participant) thresholds within the Low condition.

### 2.3. Statistical analysis

Paired sample t-tests were used to examine the effects of threat on EDA and self-reported measures of fear, anxiety, confidence and stability. In Experiment 1, paired sample *t*-tests were also used to compare RMS quotient and cross-correlation measures of actual and perceived movement. In cases where data were not normally distributed as determined by the Shapiro Wilks test, a non-parametric Wilcoxon Signed Ranks Test was used to compare height effects. In Experiment 2 and 3, a 2 (height) x 2 (stimulus characteristic) x 2 (staircase-direction) repeated measures ANOVA was used to test the effects of height (Low, High), stimulus characteristic (Experiment 2: dorsi-flexion and plantar-flexion; Experiment 3: 3 Hz and 40 Hz), and staircase-direction (ascending, descending) for calculated thresholds. If a significant stimulus characteristic effect was observed, separate 2 (height) by 2 (staircase-direction) repeated measures ANOVA’s were used to examine each stimulus characteristic independently. All dependent measures were analyzed separately (similar to previous research; Cleworth et al., 2019). The criteria for a significant result was set at *p* ≤ 0.05 with trends identified when *p* ≤ 0.1, and effect sizes reported using Cohen’s d for t-tests and partial eta squared ( $n_{p}^{2}$ ) for ANOVAs.

## 3. Results

### 3.1. Effect of height

#### 3.1.1. Emotional state

Postural threat had a significant effect on all psychological variables and EDA that was consistent across all three experiments (Table 1). Balance confidence and perceived stability decreased, while fear, anxiety and EDA increased when standing in the High compared to Low threat condition (Table 1).

#### 3.1.2. Baseline measures

There was a significant increase in vertical force at height under the stimulated (left) foot in all three experiments (< 5%, Table 1). In contrast, there was no evidence in either experiment of any effect of height on the background activity for SOL (Table 1). Similarly, there was no effect of height on the background activity for TA in Experiment 2; however, there was a significant increase at height in Experiment 1 and a non-significant trend for larger TA activity at height in Experiment 3 (Table 1). There was no effect of height on the pre-stimulus location of the foot segment or ankle angle in Experiment 2 and 3, or pre-stimulus probe force in Experiment 3 (Table 1; note, no pre-stimulus positions were calculated for Experiment 1 due to continuous perturbations used).

#### 3.1.3. Effect of height on tracking (Experiment 1)

**Figure 2:**
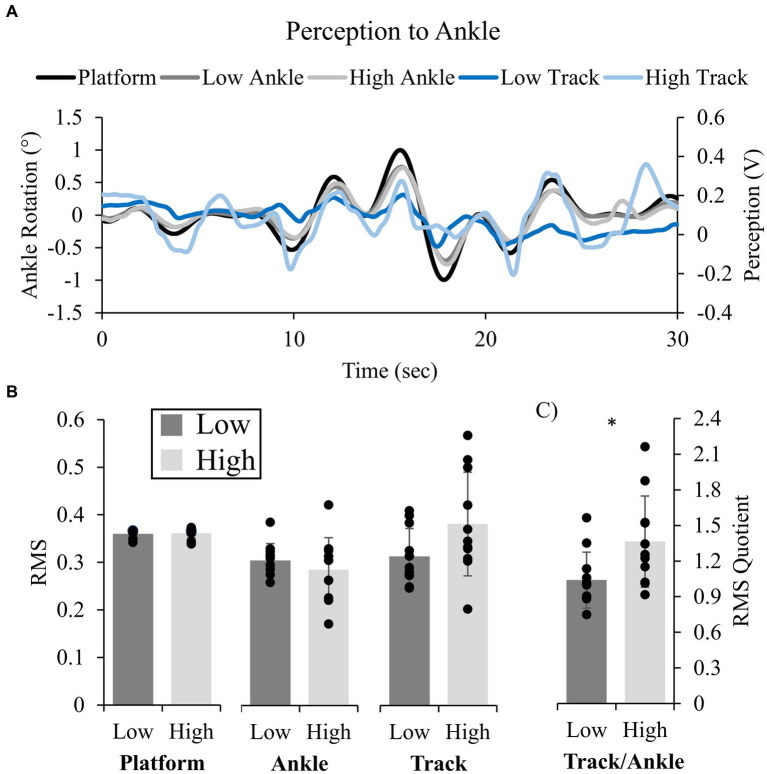
Representative traces and group mean data for Experiment 1. Platform (black), Ankle displacement (gray), and tracked displacement (blue) for Low (darker shades) and High (lighter shades) conditions **(A)**. Group mean (standard deviation) for amplitude of platform, ankle, and tracked (Track) displacements **(B)**, and calculated quotients (QRMS) between perceived and ankle motion [**(C)**; Low: black, High: gray]. Statistics were performed on platform RMS and RMS quotient only. * indicates a significant difference (*p* < 0.05).

Participants were accurate in tracking their ankle movements for both Low and High threat conditions (mean r ± SD: 0.52 ± 0.15 Low and 0.51 ± 0.14 High), which were tightly coupled to the rotations of the support surface (Figure 2A). Cross-correlations revealed no change in r (t(10) = 0.248, *p* = 0.809, d = 0.14) or lag (0.136 s ± 0.125 s Low and 0.183 s ± 0.148 s High; t(10) = 1.868, *p* = 0.091, d = 0.083) across height conditions. Similar patterns, changes in amplitude and frequencies were observed between the platform displacement, foot angular displacement and tracked movement across participants (Figure 2A).

QRMS was significantly influenced by height; QRMS increased in the High (1.36 ± 0.38) compared to Low (1.04 ± 0.24) condition, indicating more movement was perceived at height (t(10) = 2.543, *p* = 0.029, d = 0.77; Figure 2C). There were no differences in platform RMS across height conditions (Figure 2B).

#### 3.1.4. Effect of height on discriminatory thresholds (Experiment 2 and 3)

**Figure 3:**
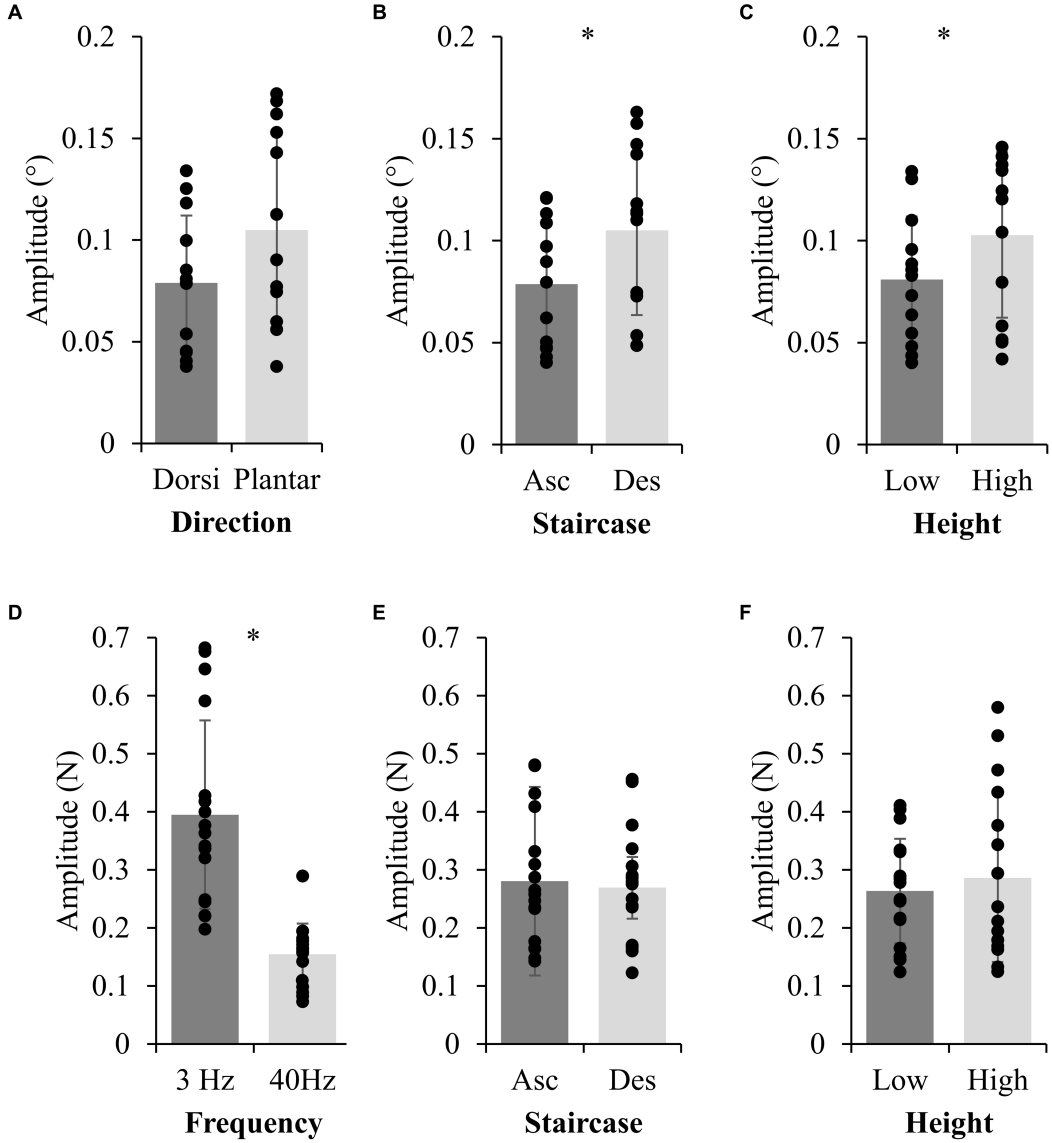
Group data for main effects on ankle rotation and foot-sole vibration thresholds. Group mean (standard deviation) from Experiment 2 (top row) and 3 (bottom row) for stimulus effects (left column), staircase direction (middle column; Asc=Ascending; Des=Descending), and height conditions (right column). * indicates a significant difference (*p* < 0.05).

In Experiment 2, there was a significant effect of height on perceptual thresholds calculated for ankle rotations (Figure 3C; *F*(1,12) = 7.285, *p* = 0.018, $n_{p}^{2}$ = 0.38). Specifically, higher perceptual thresholds across stimulus and staircase-direction were observed in the High (0.103° ± 0.041°) compared to Low condition (0.081° ± 0.031°). Nine of thirteen participants had an average increase in threshold amplitude across stimulus-direction by staircase-direction conditions. Twelve of thirteen participants had a higher threshold at height within at least two of the four stimulus-direction by staircase-direction conditions. There were no significant stimulus-direction or staircase-direction by threat interactions, or three-way interaction effects for ankle rotation perceptual thresholds. Detection thresholds for ankle rotations were on average 0.09° ± 0.03°, similar to previous work when taking into account the velocity used in the current study (Fitzpatrick and McCloskey, 1994).

In Experiment 3, there were no significant height effects on the thresholds calculated for 3 Hz (*F*(1,16) = 1.369, *p* = 0.259, $n_{p}^{2}$ = 0.08) or 40 Hz (F(1,16) = 0.137 *p* = 0.716, $n_{p}^{2}$ < 0.01) foot sole vibrations (Figure 3F). There were no significant staircase-direction by threat interactions for foot sole vibrations.

### 3.2. Effect of staircase-direction and stimulus in Experiment 2 and 3

In Experiment 2, a negative hysteresis effect was visually observed for half the trials, where the first reversal point in ascending data was smaller than the first reversal point in descending data. Participants could perceive platform-triggered ankle rotations as small as 0.03°, on average (averages ranged from 0.03° to 0.17° for ankle dorsi-flexion and plantar-flexion across heights, Figure 3A, while individual thresholds ranged from 0.024° to 0.368°). There was a significant effect of staircase-direction (F(1,12) = 9.289, p = 0.01, $n_{p}^{2}$ = 0.44), where ascending stimuli (0.079° ± 0.03°) were significantly smaller than descending stimuli (0.105° ± 0.04°) further supporting a negative hysteresis effect (Figure 3B). The counterbalance of ascending or descending stimuli resulted in no differences between first block and second block of staircase delivery (F(1,12) = 0.042, *p* = 0.840, $n_{p}^{2}$ < 0.01). There was no significant difference (F(1,12) = 3.902, *p* = 0.07, $n_{p}^{2}$ = 0.25; Figure 3A) in amplitude of detection thresholds for dorsi-flexion (0.079° ± 0.03°) compared to plantar-flexion (0.105° ± 0.05°, Figure 3A).

In Experiment 3, a perceptual hysteresis effect was visually observed, where the first reversal point in ascending data was larger than the first reversal point in descending data. There was no main effect of staircase-direction (3 Hz: F(1,16) = 1.378, *p* = 0.258, $n_{p}^{2}$ = 0.08; 40 Hz: F(1,16) = 0.155, *p* = 0.699, $n_{p}^{2}$ < 0.01) nor were there any interactions (Figure 3E). There were significant differences between the 3 Hz and 40 Hz stimuli (F(1,16) = 62.986, *p* < 0.001, $n_{p}^{2}$ = 0.80) with smaller amplitude vibrations perceived for the 40 Hz compared to the 3 Hz vibration (Figure 3D).

## 4. Discussion

The aim of this study was to determine the effect of postural threat on conscious perceptions and detection thresholds for somatosensory stimuli of the lower leg during standing. When young healthy adults stood at the edge of an elevated support surface, the amplitude of tracked ankle rotation was significantly increased (Experiment 1), despite similar ankle movements. Height-induced postural threat increased perceived ankle movement by approximately 1.3 times. This observation corroborates with prior reports of increased perception of whole-body sway in static and dynamics task compared to actual movements (Cleworth and Carpenter, 2016; Cleworth et al., 2018, 2019) and suggests sensory information from the ankle joint could contribute to threat-related changes in single-joint and whole-body movement perceptions.

To determine if the threat-related changes in perceived ankle rotation were due to changes in perceptual thresholds, we examined just-noticeable differences at the level of detection in passive ankle rotation in Experiment 2. Contrary to our hypotheses, the ability to perceive and discriminate ankle rotation direction was significantly reduced with threat, where increased perceptual thresholds were observed in the High compared to Low height condition.

Since perception of ankle rotations likely rely on muscle spindles and cutaneous receptors, we sought to isolate cutaneous threshold detection in Experiment 3. However, contrary to our hypotheses, detection thresholds for foot sole vibrations did not significantly change between Low and High height (Figure 3F). These observations are consistent with prior observations of unchanged cutaneous reflexes when standing at height (Horslen, 2016).

In the absence of evidence for decreased perceptual thresholds, the observed threat-related changes in perceptual gain during whole-body movements (Cleworth and Carpenter, 2016; Cleworth et al., 2018, 2019) and single limb rotations (Experiment 1) are likely mediated by changes in sensory response gain (Horak and Diener, 1994; Lim et al., 2014) in proprioceptive pathways, either at the spinal and/or supraspinal level. With postural threat, there is strong evidence of increased muscle spindle sensitivity, based on augmented amplitude and velocity-scaling responses to rapid ankle stretch (Horslen et al., 2018), and constant (Horslen et al., 2013) or decreased Hoffman reflexes (Sibley et al., 2007). Threat-related changes have also been observed for 1b reflexes which originate from Golgi tendon organs and have largely inhibitory effects on anti-gravity muscle activity (Horslen et al., 2017), although their role in controlling static balance is less clear. Although there is the potential for cutaneous reflexes to also be amplified by arousal during gait when modulation of cutaneous input may be critical (Zaback et al., 2018), there is less evidence for cutaneous reflex gain during stance (Experiment 3 and Horslen, 2016) when its role may be less crucial. Comparing foot sole vibrations in this study to previous work is difficult given differences in probe diameter, stimulus location, postural orientation and dependent variables used (displacement or force); however, the observations of lower detection thresholds for 40 Hz (0.154 N) vibrations compared to 3 Hz (0.395 N) vibrations matches previous reports (Strzalkowski et al., 2015).

The increased gain in movement perception observed in Experiment 1 and previous studies (Cleworth and Carpenter, 2016; Cleworth et al., 2018, 2019) may also be related to a change in sensory gating, where threat increases the amount of somatosensory information for large amplitude movements. Cortical areas receiving information pertaining to somatosensory stimuli can be modulated across tasks and by threatening conditions (Staines et al., 1997; McIlroy et al., 2003; Davis et al., 2011). The amount and direction of modulation has been shown to be reliant on the somatosensory information required for the task (Staines et al., 1997). Sensory gating occurs during passive movement (Staines et al., 1997) where faster movements increase gating (Rauch et al., 1985). Sensory gating also occurs during balance tasks (McIlroy et al., 2003) and when standing quietly in a threatening condition (Davis et al., 2011). Facilitation of initial cortical activity occur with kinaesthetic task demands (Staines et al., 1997), and with responses to destabilizing perturbations when balancing in threatening conditions (McIlroy et al., 2003; Adkin et al., 2008; Sibley et al., 2010), while later cortical processing is affected by height-induced threat (Horslen, 2016).

Selective attention is another possible mechanism for a threat-related change in perceived movement amplitude. Allocation of attentional resources to secondary tasks can directly affect performance during tactile (Lloyd et al., 1999), auditory (Haykin and Chen, 2005), visual (Verghese, 2001) and ankle-related perceptual tasks (Yasuda et al., 2014). Previous reports have indicated changes in attention can occur with height-induced threat. During static and anticipatory postural control tasks, height-induced threat increases attention toward movement-related processes, threat-related stimuli, and self-regulatory strategies while decreasing attention toward task-irrelevant information (Zaback et al., 2016). Threat-related changes in attention have also been linked to an increased conscious control and monitoring of movement (Huffman et al., 2009). These attentional changes were linked to a focus of attention during postural tasks independent of secondary tasks. Therefore, when threatened, attentional resources may be redirected to the movement perception task increasing the response gain and modifying the amplitude of perceived movements.

Based on signal detection theory (Green and Swets, 1989), the ability to detect ankle rotations or foot sole vibrations depends on the stimulus signal exceeding the current level of noise within the respective sensory modality. Since the threshold amplitude of perceived ankle rotations from the rotating platform (maximum across participants is 0.36°) were well within the ankle angular displacement from quiet standing postural tasks (1–1.5°, Gatev et al., 1999; Gage et al., 2004), height-related increases in ankle thresholds could be due to increased sensory noise created by increases in frequency of sway (Carpenter et al., 2001; Davis et al., 2009; Cleworth et al., 2012) and increased activity of proprioceptive receptors, such as muscle spindles (Horslen et al., 2013, 2018). Further evidence can be drawn from the increased false detection rates observed in Experiment 2, which has been shown to vary with the level of noise within the system (Ferrè et al., 2016).

Alternatively, the observed increase in proprioceptive detection thresholds could be related to changes in cortical involvement. Threat has the potential to influence synchronous spiking activity within and across specific cortical regions which has been associated with increased performance in consciously detecting stimuli (Melloni et al., 2007). Gamma band (40–80 Hz) oscillations are associated with attending to relevant stimuli, while alpha band (8–14 Hz) oscillations are related to the suppression of distracting stimuli (Foxe and Snyder, 2011). However, recent evidence has shown that increased anxiety increased levels of alpha band EEG (Knyazev et al., 2004) mediated through changes in movement reinvestment (Ellmers et al., 2016), and a shift from predominantly beta band to gamma band frequencies when standing under postural threat (Zaback et al., 2022). Together these changes would predict a decrease in detection thresholds (Garcia-Garcia et al., 2010), in contrast to the increased or unchanged thresholds observed in Experiment 2 and 3, respectively. However, the potential effect of attention cannot be overlooked, as simultaneous performance of a postural task (remain upright) and a perceptual task with similar stimuli to the balance task (ankle rotation) could contribute to elevated perceptual thresholds (Experiment 2).

Changes in muscle activity may also potentially contribute to altered movement-related thresholds (Taylor and McCloskey, 1992; Peters et al., 2017). There was an increase in TA activity in Experiment 1 as reported previously in studies where subjects stood facing the edge of the platform (Carpenter et al., 2001; Zaback et al., 2021). However, there were no significant changes in muscle activity observed in Experiment 2 and 3, likely due to the fact the subject was oriented perpendicular to the platform edge to control for potential confounding effects of leaning Naranjo et al., 2016).

### 4.1. Limitations

Due to the use of uncalibrated units for the tracking data, the accuracy of the amplitude of perceived movements with respect to actual movements for a given participant is difficult to identify in the current study, i.e., participants may have been underestimating ankle rotation when in the Low condition. However, given a within-subject design was used, it is clear there was a change in amplitude of perceived movement in Experiment 1, which supports previous work, indicating postural threat amplifies the perceived movement associated with ankle rotations and whole-body motion.

A change in vertical force or an increase in SOL and TA co-contraction at height may influence the detection of ankle rotations and foot sole vibrations. Vertical force on the stimulated leg did increase with height; however, this relatively small (< 5%) and potentially functionally irrelevant change is unlikely to explain the observed results, given that previous reports have illustrated no change in ankle angular displacement thresholds (Refshauge and Fitzpatrick, 1995) or foot sole vibrations (especially with 3 Hz and 40 Hz vibrations, Germano et al., 2016; Mildren et al., 2016) when standing (full body weight, large vertical force acting on foot sole) compared to lying (no vertical force acting on foot sole).

Hysteresis, judgment uncertainty, and task specificity may explain some of the results of the current study. Judgment uncertainty, which arises when participants cannot clearly separate two perceptual alternatives (Hock and Schöner, 2010), may explain the ‘negative hysteresis’ (Lopresti-Goodman et al., 2013) observed in Experiment 2. During a postural task, it may be difficult to perceive a passive ankle rotation, but not an externally produced foot sole vibration. Therefore, the negative hysteresis observed in Experiment 2 is likely mediated by uncertainty between dorsi– and plantar-flexion rotations intermixed with natural sway movements.

Finally, while upright quiet stance typically involves bilateral ankle rotations, unilateral support surface rotations were used to reduce the likelihood of evoking balance perturbations or corrective responses (Corna et al., 1996; Horslen et al., 2018), which would potentially confound the ankle somatosensory perceptual task. In addition, given these results are in line with previous work using whole body movements (Cleworth and Carpenter, 2016 Cleworth et al., 2018, 2019), these results are functionally relevant to postural control and would suggest similar results when using single vs. bilateral stimuli. However, further work is needed to address this effect.

### 4.2. Conclusion

In conclusion, perceived ankle rotation increased in amplitude, detectable thresholds for ankle rotations also increased, whereas thresholds for foot sole vibrations remained unaffected during threatening conditions. Changes in sensory receptors, afferent inflow and cortical activity are possible mechanisms to explain these results. Since perceptual thresholds in balance-relevant somatosensory systems remain unchanged or increase with postural threat, the height-related changes in perceptual gain of whole-body movements are likely attributed to stimulus gain of other sensory systems not tested in this study (e.g., vestibular or visual), or increases in response gain (increased response strength proportional to stimulus intensity; Horak and Diener, 1994; Lim et al., 2014) of all, or select balance-related sensory stimuli.

## Data availability statement

The raw data supporting the conclusions of this article will be made available by the authors, without undue reservation.

## Ethics statement

The studies involving human participants were reviewed and approved by University of British Columbia Clinical Research Ethics Board. The patients/participants provided their written informed consent to participate in this study.

## Author contributions

TC and MC contributed to conception and design of the study. TC collected and analyzed the data and wrote the first draft of the manuscript. TC and MC performed the statistical analysis. All authors contributed to the article and approved the submitted version.

## Funding

This project was supported by Natural Sciences and Engineering Research Council of Canada (NSERC) Discovery Grants for MC (326910) and TC (RGPIN-2020-06078).

## Conflict of interest

The authors declare that the research was conducted in the absence of any commercial or financial relationships that could be construed as a potential conflict of interest.

## Publisher’s note

All claims expressed in this article are solely those of the authors and do not necessarily represent those of their affiliated organizations, or those of the publisher, the editors and the reviewers. Any product that may be evaluated in this article, or claim that may be made by its manufacturer, is not guaranteed or endorsed by the publisher.
